# Keratin 17 upregulation promotes cell metastasis and angiogenesis in colon adenocarcinoma

**DOI:** 10.1080/21655979.2021.2010393

**Published:** 2021-12-22

**Authors:** Ran Ji, Yifei Ji, Lin Ma, Sijia Ge, Jing Chen, Shuzhen Wu, Tianxin Huang, Yu Sheng, Liyang Wang, Nan Yi, Zhaoxiu Liu

**Affiliations:** a Department of Gastroenterology, Affiliated Hospital of Nantong University, Nantong, China; b Research Center of Clinical Medicine, Nantong University, Affiliated Hospital of Nantong University, Nantong, China; c Department of Gastroenterology, Affiliated Haian Hospital of Nantong University, Nantong, China

**Keywords:** Colon adenocarcinoma, KRT17, β-catenin, metastasis, angiogenesis

## Abstract

Colon adenocarcinoma (COAD), having high malignancy and poor prognosis, is the main pathological type of colon cancer. Previous studies show that Keratin 17 (KRT17) plays an important role in the development of many malignant tumors. However, its role and the molecular mechanism underlying COAD remain unclear. Using TCGA and ONCOMINE databases, as well as immunohistochemistry, we found that the expression of KRT17 was higher in COAD tissues as compared to that in the adjacent normal tissues. Cell- and animal-based experiments showed that overexpression of KRT17 promoted the invasion and metastasis of colon cancer cells while knocking down KRT17 reversed these processes both *in vitro* and *in vivo*. In addition, we also showed that KRT17 promoted the formation of new blood vessels. Mechanistically, KRT17 could regulate the WNT/β-catenin signaling pathway, and APC may be involved in this process by interacting with KRT17. In summary, these findings suggested that high expression of KRT17 could promote cell metastasis and angiogenesis of colon cancer cells by regulating the WNT/β-catenin signaling pathway. Thus, KRT17 could be a potential therapeutic target for COAD treatment.

## 1. Introduction

Colon adenocarcinoma (COAD) is the main pathological type of colon cancer and globally, is the second deadliest cancer, killing an average of 900,000 people every year. Although the human life span has extended due to the improvement in medical examinations , the incidence of COAD is still on the rise [1]. COAD usually develops first from colon polyps and eventually transforms into malignant tumors owing to a series of changes in the tumor microenvironment and gene mutations [2]. Tumor metastasis and angiogenesis further accelerate the progression of the disease and result in an extremely poor prognosis [3]. Therefore, the identification of new cancer molecular markers as novel targets for COAD is an important need for future clinical treatment.

Keratin is a member of the intermediate-filament-forming protein superfamily. It is classified into type I and type II protein, both of which are expressed in epithelial cells in the form of intermediate filaments [4]. Previous studies show that keratin activation is involved in the metastasis and invasion of cancer cells [5,6]. Keratin17 (KRT17), also known as cytokeratin 17 (K17), is a type I intermediate filament, which regulates several biological processes [4], including cell proliferation and growth, immune response, and the differentiation of skin appendages [7–9]. Recently, many studies report the critical role of KRT17 in many malignancies, including cervical cancer [10], basal skin tumor [11], urothelial tumor [12], pancreatic cancer [13], and gastric adenocarcinoma [14]. Although, it has been reported that the expression of KRT17 in colon cancer tissues was higher than that in normal colonic epithelial tissues, and increased concomitantly with the grade of T staging progresses [15]. In addition, Daisuke Ujiie et al. has validated that the expression of KRT17 can discriminate postoperative stage II patients who are at high probability of disease recurrence [16]. However, the role of KRT17 in the biological behavior of colon cancer cells and related molecular mechanisms remain unclear.

Given the previous findings, we hypothesized that KRT17 plays a critical role in the development of COAD. Interestingly, we found that KRT17 is highly expressed in COAD tissues and cell lines, and could promote the cells metastasis and angiogenesis *in vivo and in vitro*. Through GSEA analysis, we found that KRT17 could affect the progression of COAD through the WNT/β-catenin signaling pathway. Therefore, in this study, we aimed to explore the effects of KRT17 on cell metastasis and angiogenesis in COAD and the findings may have implications in the search for new therapeutic targets.

## 2. Materials and methods

### 2.1 Data acquisition from TCGA, ONCOMINE, and STRING databases

Oncomine: KRT17 expression profiles in colon tumor and adjacent normal tissues were collected and analyzed using the Oncomine online database (http://www.oncomine.com). A dataset of the known gene expression patterns was included to validate the mRNA expression of KRT17 in the two groups. An unpaired t-test was used to analyze the significant differences.

TCGA: A total of 481 COAD and 41 adjacent normal tissue samples along with their corresponding RNAseq data were downloaded from The Cancer Genome Atlas (TCGA) database and XENA (https://xenabrowser.net/heatmap/) [17]. The gene expression profile was experimentally determined on the Illumina HiSeq 2000 RNA Sequencing platform of the University of North Carolina TCGA genome characterization center. The data were in FPKM format. Student t-tests were used to compare differential transcript level expressions of KRT17 between the paired tumor and normal tissues TNM staging and grade.

STRING: Search Tool for the Retrieval of Interacting Genes (STRING; http://string-db.org)(version 10.0) online database was used to predict the PPI network of KRT17-correlated hub genes and analyze the functional interactions between proteins. Interactions with a combined score > 0.4 were considered statistically significant.

### 2.2 Clinical tissue samples

All COAD and corresponding adjacent normal tissues were obtained from the Nantong University Hospital. A total of 78 COAD tumor samples and 20 tumor and corresponding adjacent non-tumor tissue pairs were collected from patients between 2018 to 2020 (Supplementary EXCEL 1). All patients who did not receive radiation, chemotherapy, hormones, or related anti-tumor therapies before surgery, and had no other cancer diagnoses, were included in our analyses. Follow-up patient information was collected through outpatient clinics or telephonic conversations every six months or until their death. Clinical information was provided by the patients or their relatives. Written consent from patients or their relatives was obtained and all clinical samples and experimental procedures were approved by the Human Research Ethics Committee of the Affiliated Hospital of Nantong University (82,000,497).

### 2.3 Gene Set Enrichment Analysis (GSEA)

GSEA (version 4.1.0) package was used. For each separate analysis, the statistical student’s t-test score was obtained for the pathway with consistent expression; the mean differential expression of each gene was calculated. A permutation test was performed 1000 times to identify the pathways with significant changes. The patients were divided into the high KRT17 and low KRT17 expression groups according to the KRT17 expression information in the TCGA-COAD cohort.

### 2.4 Real-time PCR

We used TRIzol reagent to extract the total cell and tissue mRNA and reverse-transcribed the mRNA to complementary DNA. We used SYBR Premix Ex Taq and 7500 real-time PCR to determine the mRNA expression of KRT17 in real-time PCR. Primer designs for KRT17 were as follows: forward CTCCTCCCAGAGGAAGAACTGG and reverse TCTTGAGTCCTCTCTGCGTG. GAPDH was used as the internal reference and the primer designs were as follows: forward CAGGAGGCATTGCTGATGAT and reverse GAAGGCTGGGGCTCATTT.

### 2.5 Nuclear and cytoplasmic protein extraction

After the appropriate treatment, the nuclear and cytoplasmic fractions of proteins were extracted from the cells. Briefly, cells were treated with 250 μL extraction buffer (10 mmol/L Tris-HCl, 10 mmol/L KCl, and 5 mmol/L MgCl2; pH 7.6). Then, to disrupt the cell membrane, cells were incubated with 0.5% Triton X-100 for 40 min. 250 μL of nuclear isolation buffer (10 mmol/L Tris-HCl, 10 mmol/L KCl, 5 mmol/L MgCl2, and 0.35 mol/L sucrose) was subsequently added, and the sample was subjected to density gradient centrifugation for 10 min. The supernatant contained the cytoplasmic fraction and the precipitate contained the nuclear fraction. The supernatant was transferred to a fresh centrifuge tube, and four volumes of pre-chilled acetone were added to it at −20°C and incubated overnight. Next, the supernatant was centrifuged at 12, 000 rpm for 20 min at 4°C. The precipitate was dissolved in SDS buffer and centrifuged at 12, 000 rpm for 30 min at 4°C. After centrifugation, the supernatant containing the nuclear proteins was collected.

### 2.6 Western blotting

The total cell protein was extracted and incubated with protein lysate buffer (Biyuntian, China). The proteins were transferred onto a polyvinylidene fluoride membrane (Millipore, USA) after separation by 10% SDS-PAGE. The membrane was blocked with 5% skim milk for two hours and then incubated with the corresponding primary antibody overnight at 4°C. Subsequently, the membrane was incubated with a diluted secondary antibody for two hours. Finally, the ECL luminescent solution was evenly spread onto the PVDF membrane and the blot was developed. The antibodies used were as follows: KRT17 (1:1000, Proteintech, China), β-catenin (1:1000, Proteintech, China), APC (1:1000, Boster, China), and GAPDH (1:5000, Santa Cruz Biotechnology, USA),Lamin A/C(1:2000, Santa Cruz Biotechnology, USA). GAPDH was used as a loading control. Image J software was used to quantify the band intensities.

### 2.7 Co-immunoprecipitation (Co-IP)

SW116 cells (2 × 10^6^) were harvested and lysed using a lysis buffer (50 mM Tris-HCl pH 7.5, 150 mM NaCl, 1 mM EDTA, 0.3% Triton X-100, and 1 mM protease inhibitor PMSF) on ice for 30 min. The cell lysate was centrifuged at 4°C at 12, 000 rpm for 30 min; the supernatant was collected. A small amount of lysate was taken for Western blot analysis. 1 μg of the corresponding antibody was added to the remaining lysate and incubated by slow shaking overnight at 4°C.10 μl of protein A agarose beads were washed thrice with an appropriate amount of lysis buffer and centrifuged at 3,000 rpm for three minutes each time. The pretreated 10 μl protein A agarose beads were added to the cell lysate and incubated by slow shaking at 4°C for two to four hours to allow the coupling of the antibody to the protein A agarose beads. The precipitates were washed eight times with ice-cold lysis buffer, resuspended in the PBS, and resolved by SDS-PAGE followed by Western blotting.

### 2.8 Inmunohistochemistry (IHC)

Samples were processed for immunohistochemical analysis to determine the KRT17 and CD31 expression levels and their localization. Rabbit polyclonal antibodies KRT17 (1:500, Proteintech, China) and CD31 (1:200, Proteintech, China) were used for detection. The antigen-antibody complex was visualized using diaminobenzidine (DAB, 5 minutes incubation) and counterstained with hematoxylin. PBS was used as the negative control. KRT17 immunoreactivity was detected in the cytoplasm of the carcinoma cells, CD31 was detected in the neovascularization, and the sample sections were scored semi-quantitatively for immunoreactivity as follows: 0 = 0% stained; 1 = 1–49% stained; 2 = 50–100% stained immunoreactive cells. Additionally, the intensity of staining was scored semi-quantitatively as 0, negative; 1, weak; 2, intermediate; and 3, strong. The final immunoreaction score was defined as the sum of both extent and intensity parameters. Final immunoreaction scores >0 were defined as positive. Photographs were observed under an optical microscope. Two experienced pathologists independently evaluated the slices.

### 2.9 Immunofluorescence staining

To determine the localization of β-catenin and KRT17, cells were seeded on coverslips and fixed using 4% paraformaldehyde for 40 min. Coverslips were rinsed with PBS, and cells were permeabilized with 0.1% Triton X-100 for 20 min. Following PBST washes thrice, the cells were blocked using 1% BSA at 4°C for two hours. Subsequently, the cells were incubated with anti-β-catenin antibody (1:200, Proteintech, China) and anti-KRT17 antibody overnight at 4°C. After washing with PBST, cells were incubated with rhodamine-labeled goat anti-rabbit secondary antibody (1:200, Sigma, USA) for 1 h and 40,6-Diamidino-2-phenylindole (DAPI; Invitrogen, USA) was used to stain the nucleus. Images were captured using the OLYMPUS U-RFL-T upright fluorescence microscope.

### 2.10 Angiogenesis assay

For the test tube formation assay, the Matrigel matrix (Corning, USA) was incubated at 37°C for 30 minutes to polymerize the Matrigel in a 24-well plate such that the Matrigel was flat and free of bubbles. HUVEC cells treated with COAD supernatant were seeded into Matrigel-coated wells. The plate was then incubated at 37°C in a humidified atmosphere containing 5% CO_2_. The formation of the tube was observed with a Nikon inverted microscope at 12 hours. The tubule forming ability was determined by measuring the number of tubes. All assays were performed in triplicates and each experiment was repeated thrice.

### 2.11 CAM assay

The chicken embryo CAM angiogenesis model was used to study angiogenesis in vivo. A circular window with a diameter of approximately 5 mm was created on the egg of an 8-day-old chick embryo, which could communicate with the chorioallantoic membrane (CAM) below it. A 1 ml syringe was used to inject 200 μl sterile colon cancer cell supernatant containing approximately 5,000 cells and the window was subsequently covered with sterile tape similar to the procedure described previously [18]. The eggs were placed into a sterile incubator, incubated at 37°C for 48 hours, and collected. The CAM was fixed in situ. Briefly, it was cut out from the egg, placed on a glass slide, and using a high-definition imaging digital camera, the CAM was photographed. Thus, the growth of blood vessels was quantified. We collated the data into a Microsoft Excel spreadsheet and calculate the means and variances.

### 2.12 Matrigel plug angiogenesis assay

The assay was performed according to the previously described method [19]. Briefly, 0.5 ml of Matrigel containing sterile cell supernatant and control samples were subcutaneously injected into the ventral area of eight-week-old C57BL/6 male mice. After 10 days, the mice were sacrificed and the Matrigel plugs were taken out intact, fixed with 3% formalin solution, embedded, sliced, and stained by three-color staining technique. The formation of blood vessels was observed through microscopic observation of the slices. The Animal Resources Committee of the Nantong University approved the protocols of animal research.

### 2.13 Cell culture and transfection

Human colon cancer cell lines, SW116, LOVO, SW480, RKO, SW620, HCT116, HT29, and human umbilical vein endothelial cells (HUVEC) were obtained from the Immunology Laboratory of Nantong University School of Medicine. These were verified by karyotyping. The cells were cultured in DMEM supplemented with 10% FBS and 1% penicillin/streptomycin. The cells were grown in sterile Petri dishes and passaged every three days with 0.25% trypsin. SW116 and LOVO cells were seeded onto a six-well plate 24 hours before transfection. Overexpression and empty vector control plasmids were purchased from (Shanghai Xi Hui Biotechnology Co., Ltd., China). Small interfering RNA (siRNA) and control siRNA against KRT17 were synthesized. Cells were transfected with plasmid or siRNA following the manufacturer’s instructions using Lipofectamine® 2000 (Invitrogen, USA).

### 2.14 Wound healing assay

48 hours after the cells were transfected, about 2 × 10^5^ cells were seeded into six-well plate, and incubated at 37°C until reaching 100% confluency.Then,an artificial wound was created by scraping cells with a sterile 200 μl pipette tip. Cells were then washed with 1× PBS to remove floating debris. After scratching, a Nikon inverted microscope was used to photograph the scratched area at 100x magnification at 0 h and 24 h after gaps were generated. The cell migration rates were calculated by using the ImageJ software (Version 1.47 v).

### 2. 15 Transwell assay

Cell migration and invasion were measured by Transwell assay using the 24-well Transwell chamber containing 8 μm polycarbonate membranes. By cell counting, approximately 1 × 10^5^ cells were seeded in the upper chamber containing 200 μL of serum-free medium. Then, the lower chamber was inserted into the 24 wells containing 500 μL of 10% DMEM. After incubation at 37°C for 24 hours in the cell incubator, cells were scraped off of the membrane surface. Cells were fixed with methanol and stained with 0.5% crystal violet. Each well was photographed with a Nikon inverted research microscope and counted in not less than three random 100x microscope fields.

### 2.16 Tail vein metastasis and intravital imaging assay

According to the previously described experimental method [20], six-week-old male nude mice purchased from the Clinical Animal Research Center of Nantong University were used for the standard tail vein metastasis assay. The cells were trypsinized, approximately 1 × 10^6^ cells were counted, the single-cell suspension was injected into the tail vein with a scalp needle, and the nude mice were maintained for six weeks in a sterile environment. The mice were anesthetized with ketamine and 150 mg/kg fluorescein was injected intraperitoneally. After anesthetizing, the nude mice were put into a dark box of the small animal multispectral imaging system and recorded. A photographic gray-scale image was taken, and the bioluminescence signal was displayed in pseudo-color and projected onto the gray-scale image using Simple PCI software.

### 2.17 Data analysis

Statistical analyses were performed using GraphPad Prism8 and SPSS statistical software. Unless otherwise stated, all data were determined by three independent experiments and expressed as mean ±SD. The data sets between sample groups and within samples were compared by analysis of variance; P < 0.05 was considered statistically significant. For graphic representation, Adobe Illustrator CC, Adobe Photoshop CC, and Image J software were used.

## 3. Results

By mining databases such as TCGA, interestingly, we found that KRT17 was highly expressed in COAD tissues, and the findings were experimentally verified by immunohistochemical staining. To understand how KRT17 affected the progression of COAD, we performed several experiments *in vivo and in vitro* and found that increased expression of KRT17 could promote the invasion and migration of colon cancer cells. Simultaneously, it impacted the formation of new blood vessels. In addition, through the GSEA, we found that KRT17 was related to the WNT signaling pathway, and Wnt3a, an activator of WNT signaling, could potentially promote the expression of KRT17. By STRING analysis, we found that APC genes could interact with KRT17, and the correlation between the two genes was verified by CO-IP experiments. APC overexpression had a negative regulatory effect on the migration and invasion caused by KRT17.

### 3.1 KRT17 is upregulated in COAD

**Figure 1.**
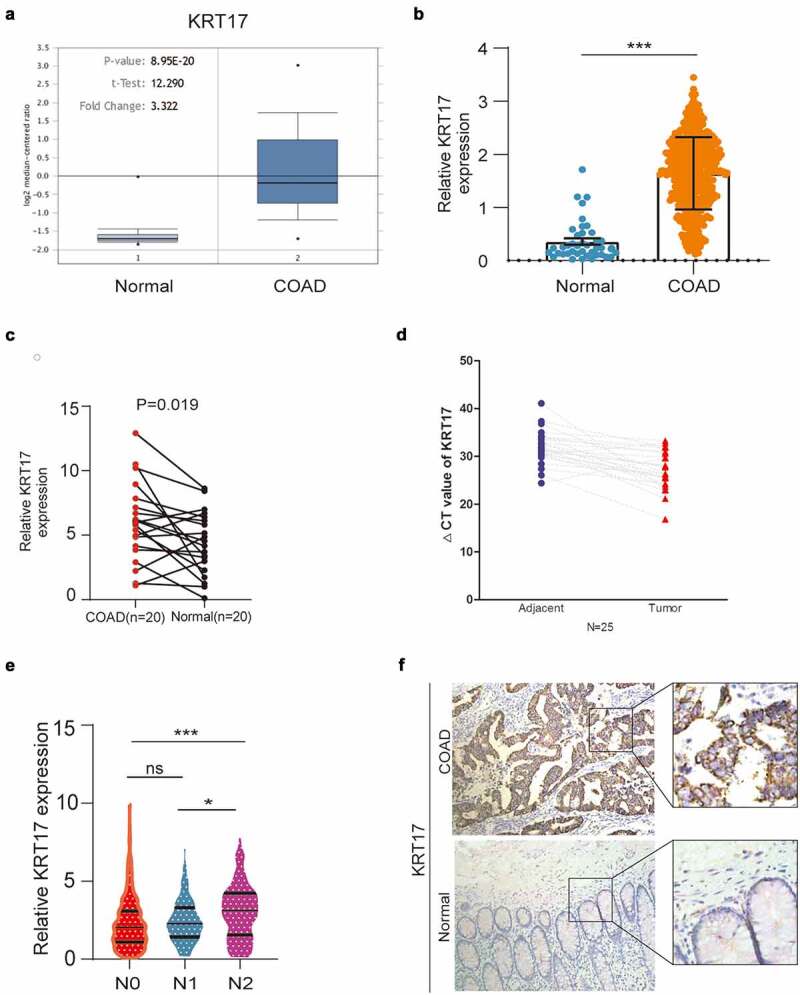
KRT17 upregulation in COAD. (a, b) Data from Oncomine and TCGA databases shows that KRT17 is upregulated in COAD as compared to the normal control tissues. (c) KRT17 expression in 20 paired COAD and matched adjacent normal tissues from TCGA database; P-values are shown (n = 20). (d) RT-PCR analysis of KRT17 expression in 25 COAD tissues; ΔCT value is inversely proportional to relative expression. (e) KRT17 expression is related to the N stage (*P < 0.05 **P < 0.01 ***P < 0.001). (f) Immunohistochemical staining for KRT17 in COAD and normal tissues

To understand the potential role of KRT17 in COAD, different databases were used to analyze the expression of KRT17. In the ONCOMINE database, the transcript levels of KRT17 in the COAD samples were 3.322-fold as compared to the normal tissues (Figure 1(a)). In the TCGA database, we found that the mRNA levels of KRT17 were significantly higher in COAD as compared to normal tissue samples (Figure 1(b)). At the same time, through the TCGA analysis of the characteristics of COAD patients, we found that the expression of KRT17 is correlated with age, tumor status, and TNM stage. But there is no correlation with other factors such as gender and tumor location(Table 1).Furthermore, in the paired carcinoma and adjacent non-tumor tissues of COAD 20 patients, we found that KRT17 mRNA levels were significantly higher in COAD tissues as compared to the adjacent normal tissues (Figure 1(c)). We further confirmed the expression of KRT17 in 25 pairs of tissue samples collected from our hospital (Figure 1(d)). In addition, from the TCGA database, we also found that the high expression of KRT17 was correlated with the regional lymph node (N) stage of the tumor (Figure 1(e)). Finally, we detected the expression of KRT17 in 10 patient tissues by immunohistochemistry and found that KRT17 was highly expressed in COAD tissues than in the adjacent normal tissues (figure 1(f) and Supplementary figure 1). These data indicated that KRT17 expression was upregulated in COAD tissues and it could play an important role in the development of COAD.

**Table 1.** Correlationship between COAD patient characteristics and KRT 17 level (*P < 0.05). – – TCGA

	KRT 17 level				
Clinicalpathological fetures	Low(n = 215)	High(n = 215)	Total	X^2^	P value
Gender				0.373	0.541
Male	109	113	222		
Female	96	112	208		
Age(years)				4.272	0.039*
≤60	44	68	112		
>60	161	157	318		
Person neoplasm cancer status				36.094	0.000*
Tumor free	33	1	34		
With tumor	172	224	396		
Tumor location				1.553	0.213
Colon	204	221	425		
Rectosigmoid junction	1	4	5		
TNM stage				5.232	0.022*
I/II	129	117	246		
III/IV	76	108	184		
T stage				5.320	0.021*
1/2	47	32	79		
3/4	158	192	350		
N stage				9.979	0.007*
0	135	118	253		
1	45	57	102		
2	25	50	75

### 3.2 KRT17 promotes migration and invasion of colon cancer cells

**Figure 2.**
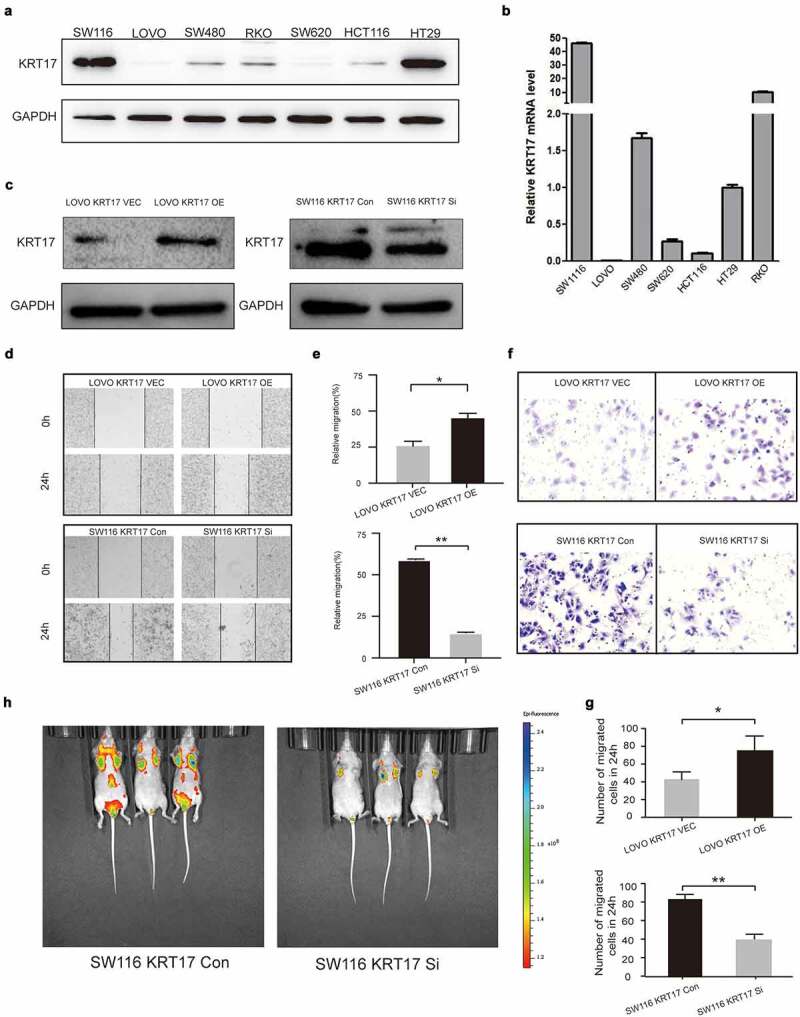
KRT17 promotes the migration and invasion of colon cancer cells. (a, b) Western blotting and RT-PCR analyses of KRT17 expression in seven colon cancer cell lines; mean ± SEM are displayed (n = 3). (c) WB analysis for the efficiency of si-KRT17 and si-Control transfection in SW116 cells; overexpression plasmid and empty control vector transfection in LOVO cells. (d) Wound healing assay for cell migration in SW116 and LOVO cells; the images of wound closure are shown for the indicated number of hours after scratching (0, 24 h). (e) The quantification for wound healing assay. (f) Transwell assays to examine the potential migration and invasion of transfected cells. (g) The quantification of (F). (h) intravital imaging assay for cell metastasis in SW116 KRT17 Con and SW116 KRT17 si cells

To further investigate the biological function of KRT17 in colon cancer cells, we measured the expression of KRT17 in seven colon cancer cell lines and accordingly selected SW116 and LOVO cell lines for subsequent cell-based experiments (Figure 2(a‒b)). LOVO and SW116 cells were transfected with KRT17 overexpression and siRNA plasmids, respectively (Figure 2(c)). Wound-healing and Transwell assays were used to analyze the effects of KRT17 on the migration and invasion of colon cancer cells. The results showed that KRT17 overexpression promoted the migration and invasion of LOVO cells while knocking down KRT17 reversed this trend (Figure 2(d-g)). Furthermore, we also constructed a tumor metastatic model by injecting SW116 luciferase cells into the tail vein of nude mice, and tumor metastasis was monitored *in vivo* using an imaging system after six weeks. The results showed that knocking down KRT17 inhibited the metastatic ability of SW116 cells (Figure 2(h)). Taken together, our data suggested that KRT17 could promote migration and invasion of colon cancer cells both *in vitro and in vivo*.

### 3.3 Overexpression of KRT17 induces angiogenesis in vivo

**Figure 3.**
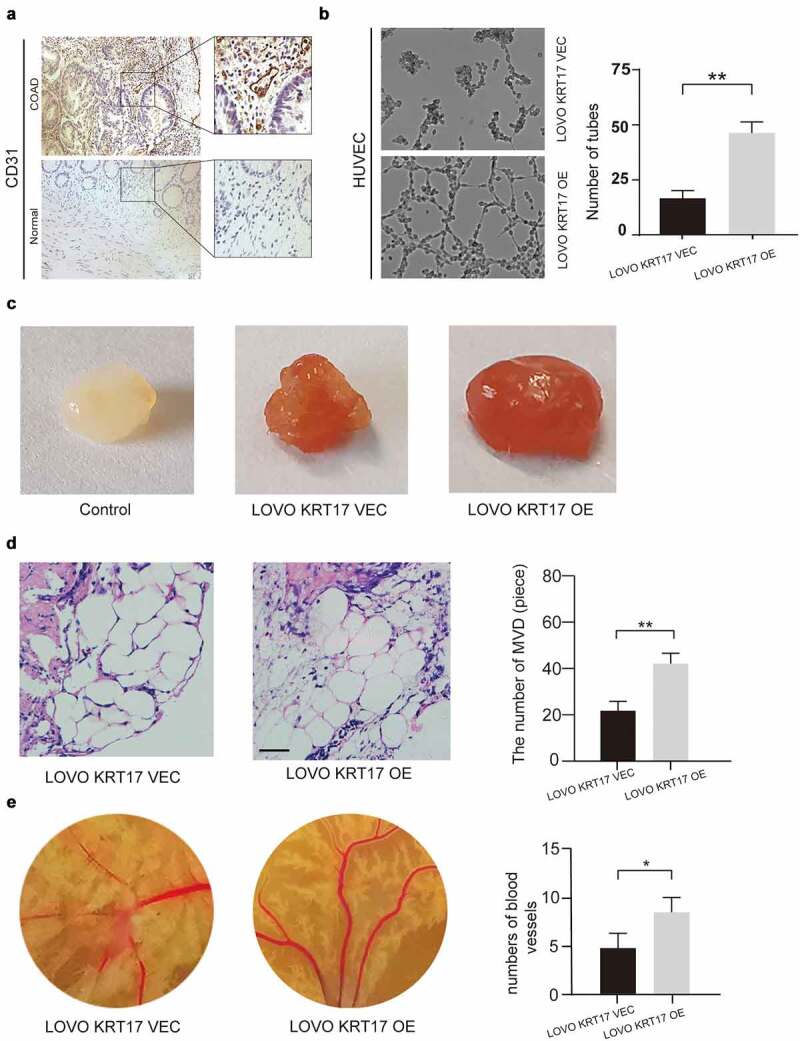
Overexpression of KRT17 induces angiogenesis in vivo. (a) Immunohistochemical staining for CD31 in COAD and normal tissues. (b) The effect of KRT17 expression on the tube formation ability of HUVEC by tube formation assay; mean ± SEM are displayed (n = 3). (c) Matrigel angiogenesis assay reflects the effect of KRT17 expression on neovascularization in comparison with the control group. (d) H&E staining of Matrigel sections and the number of MVDs; mean ± SEM are displayed (n = 3). (e) Images of the blood vessels of chick embryos; bar chart demonstrates the relative number of blood vessels; data are presented for at least three independent experiments (*P < 0.05 **P < 0.01 according to the two-tailed Student’s t-test)

Previous studies show that angiogenesis plays a vital role in the development and metastasis of COAD [21]. Thus, we speculated that KRT17 was correlated with angiogenesis in COAD. First, we detected the expression of CD31, a specific marker of endothelial cells, and found that its expression was higher in the COAD tissues than in the adjacent normal tissues (Figure 3(a) and Supplementary figure 2). Next, the tube forming assay, matrix plug assay, and chicken blastocyst assay were used to examine the potential effect of KRT17 on angiogenesis both *in vivo* and *in vitro*. The tubule formation assay showed that overexpression of KRT17 in LOVO cells could promote the *in vitro* tubule formation ability in HUVEC cells (Figure 3(b)). The matrix plug assay showed that the Matrigel plugs in the experimental group transfected with KRT17 overexpression plasmid were dark red, while these were white in the control group, which indicated that overexpression of KRT17 could promote the formation of new blood vessels in the emboli *in vivo* (Figure 3(c)). This result was further confirmed by counting the neovascularization after staining the embolized sections (Figure 3(d)). Furthermore, the chicken blastocyst assay and subsequent measurements of the chicken allantoic membrane also showed that KRT17 could promote the formation of blood vessels during the development of the chicken embryo (Figure 3(e)). These results indicated that KRT17 was correlated with angiogenesis in COAD.

### 3.4 Association between KRT17 and WNT signaling pathway

**Figure 4.**
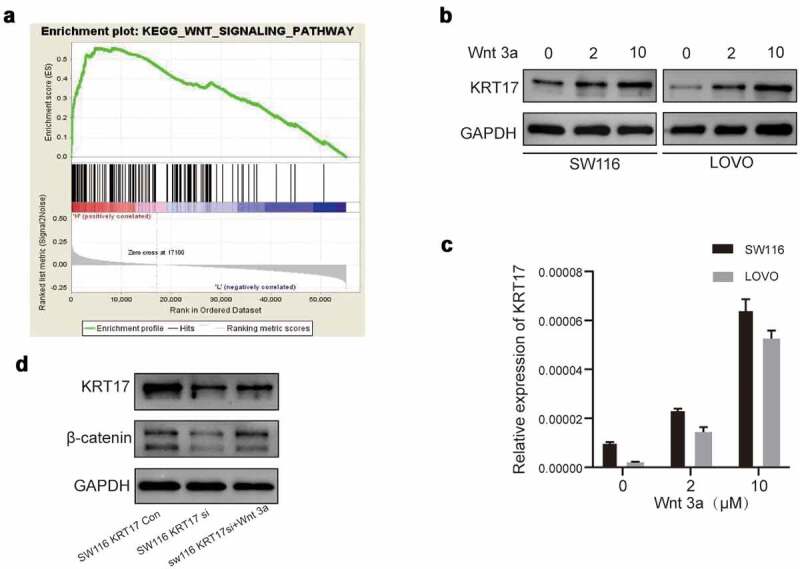
The relationship between KRT17 and WNT signaling pathway. (a) The result above meets the criteria of FDR<0.25, P-value < 0.05; high expression of KRT17 is significantly related to the WNT signaling pathway. (b, c) Western blotting and RT-PCR analyses of KRT17 expression at different concentrations of Wnt3a (0, 2, and 10 μM) treatment for 24 hours. (d) Protein level expression of Wnt/β-catenin pathway members, β-catenin and KRT17, in SW116 KRT17 Con cells and SW116 KRT17 si cells with or without Wnt3a (2 μM) pretreatment

The above results showed that KRT17 could promote migration, invasion, and angiogenesis in colon cancer cells, however, its underlying molecular mechanism remains unknown. Therefore, we performed GSEA to identify the possible signaling pathways involving KRT17 in COAD and found that the high KRT17 expression was related to the WNT signaling pathway (Figure 4(a)). As an activator of the WNT/β-catenin signaling pathway, the function of Wnt3a has been widely studied [22]. Thus, SW116 and LOVO cells were treated with different concentrations of Wnt3a for 24 hours. The Western blotting results showed that the expression of KRT17 increased with the increase in Wnt3a dose (Figure 4(b) and 4c). In addition, we also found a decrease in the expression of β-catenin in SW116 cells transfected with KRT17-siRNA, which recovered upon treatment with Wnt3a (Figure 4(d)). In order to explore the localization of β-catenin in SW116 cells, the nuclear protein and cytoplasmic protein from SW116 cells transfected with control-siRNA or KRT17-siRNA with or without Wnt3a stimulation were extracted and detected by Western blotting assay. The results showed that β-catenin was mainly distributed in the nucleus in SW116 cells (Supplement figure S3a). The level of β-catenin in the nucleus was reduced in SW116 cells transfected with KRT17-siRNA, but recovered when stimulated with Wnt3a. In addition, the nucleus distribution of β-catenin in SW116 cells was confirmed by immunofluorescence experiment (Supplement figure S3b). Furthermore, we also found that KRT17 was mainly distributed in the cytoplasmic in SW116 cells (Supplement figure S3b).These results indicated that KRT17 was indeed related to the WNT/β-catenin signaling pathway in COAD.

### 3.5 Correlation between KRT17 and APC genes

**Figure 5.**
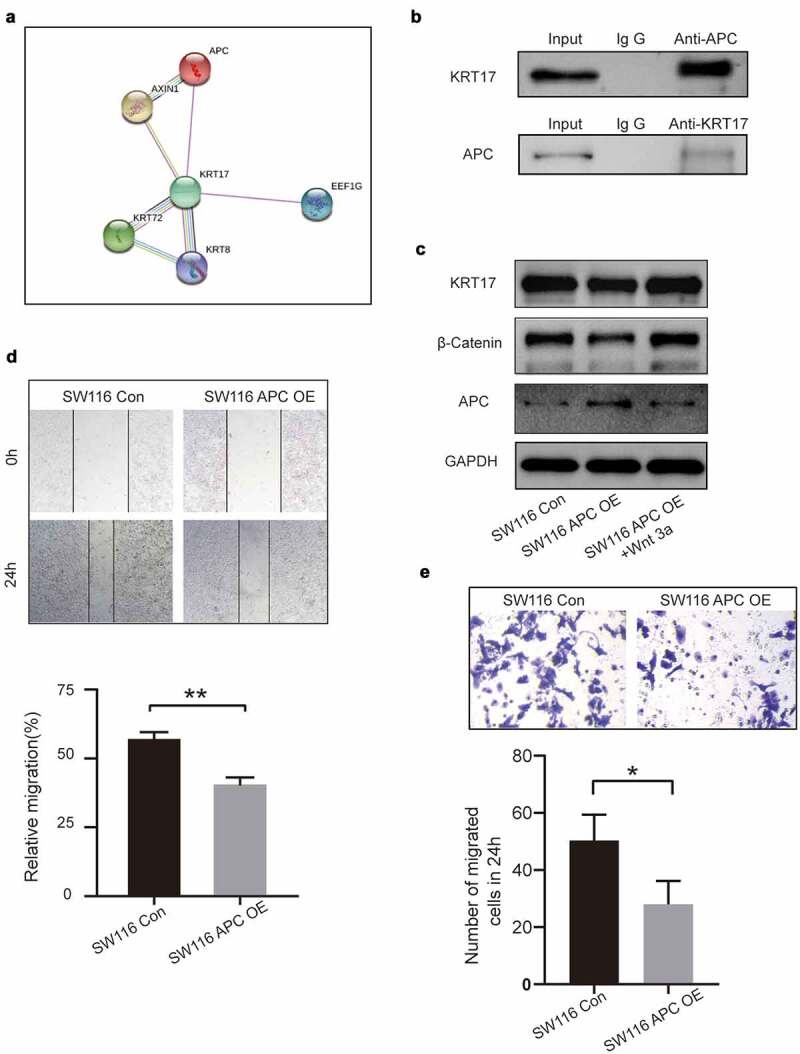
Correlation between KRT17 and APC genes. (a) Using the STRING online database PPI network of the KRT17 was constructed. (b) Co-immunoprecipitation shows that KRT17 interacts with APC in human COAD cell lines. Total cell lysate from SW116 cells was immunoprecipitated with anti-APC and anti-KRT17 antibodies. (c) Protein level expression of KRT17, β-catenin, and APC in SW116 cells. SW116 APC OE cells with or without Wnt3a (2 μM) pretreatment. (d, e) Wound healing and Transwell assays show that APC overexpression inhibits the migration and invasion of SW116 cells; mean ± SEM are displayed (n = 3) (*P < 0.05 **P < 0.01 according to the two-tailed Student’s t-test)

To further explore the effects of KRT17 in the WNT signaling pathway, the STRING online database was used to identify KRT17-related genes. We found an interaction between KRT17 and APC, a member of the WNT signaling pathway (Figure 5(a)). Subsequently, using CO-IP, we experimentally verified the correlation between KRT17 and APC (Figure 5(b)). Next, SW116 cells were transfected with APC overexpression plasmid with or without Wnt3a treatment. We found that the expressions of both KRT17 and β-catenin reduced due to the overexpression of APC, while they increased in SW116 cells treated with Wnt3a (Figure 5(c)). Additionally, we found that compared with the control group, the expression of APC reduced in SW116 cells stimulated with Wnt3a (Figure 5(c)). The results of the wound-healing and Transwell assays also showed that overexpression of APC inhibited invasion and migration in colon cancer cells (Figure 5(d-e)). These results show that in colon cancer cells, APC may be involved in the regulation of KRT17-WNT/β-catenin signaling pathway by interacting with KRT17.

## 4. Discussion

Although the knowledge on the molecular biological mechanism underlying COAD has progressed in recent years, effective treatment methods for this solid tumor having a high degree of invasion and poor prognosis are still lacking. Therefore, there is a need to identify and verify new molecular markers to accelerate the development of future treatment strategies. KRT17 is a member of type I epithelial keratin family of proteins. It is an oncogene of many malignant tumors. According to a previous proteomic study, KRT17 is highly expressed in COAD [15]. Correspondingly, this result laid the foundation for our follow-up research on the effects of KRT17 in COAD.

In this study, through Oncomine and TCGA database analyses, we found that KRT17 mRNA expression in COAD was significantly up-regulated. Interestingly, by immunohistochemical staining of COAD and its corresponding adjacent normal tissues, we found that KRT17 is almost absent in the normal colon epithelial tissues. After analyzing the information of 78 patients from the Affiliated Hospital of Nantong University, we found that the expression of KRT17 is not only related to TNM staging and survival but also the microvessel density (MVD) (Table 2). Interestingly, CD31, a marker of angiogenesis [23] reported in liver cancer and cholangiocarcinoma [24], was also detected in COAD; its expression was significantly high. Tumor invasion, metastasis, and increased angiogenesis indicate a poor prognosis of the disease [25]. In the present study, we found that KRT17 was closely related to these functions in tumor cells.

**Table 2.** Correlationship between the clinicopathological features of COAD patients and the level of KRT17 (*P < 0.05). – – Affiliated Hospital of Nantong University

	KRT 17 level				
Clinicalpathological fetures	Low(n = 39)	High(n = 39)	Total	X^2^	P value
Gender				2.731	0.098
Male	24	27	51		
Female	18	9	27		
Age(years)				0.542	0.462
≤60	21	15	36		
>60	21	21	42		
Tumor diameter(cm)				6.798	0.009
≤4	18	26	44		
>4	24	10	34		
Tumor location				0.125	0.724
Up/ Middle	17	16	33		
Down	25	20	45		
Tumor differentiation				0.489	0.484
High	20	20			
Low	22	16			
CEA level(ng/ml)				1.697	0.193
≤5	27	28	55		
>5	15	8	23		
					TNM stage				19.192	0.000
I/II	13	29	42							
III	29	7	36							
T stage				8.254	0.004					
1/2	10	20	30							
3/4	32	16	48							
Survival state				14.480	0.000					
Death	10	24	34							
Alive	32	12	44							
N stage				31.536	0.000					
0/1	17	36	53							
2/3	25	0	25							
MVD				29.534	0.000					
High	29	3	32							
Low	13	33	46							

Previous reports show that abnormal regulation of the WNT signaling pathway is crucial in cancer biology [26] and β-catenin is related to poor cancer prognosis; nuclear accumulation of β-catenin can promote the proliferation and senescence of cancer cells [27]. In a previous study, the pharmacological inhibition of PI3K-Akt signaling in COAD by over-activation of WNT signaling led to nuclear accumulation of β-catenin, which in turn resulted in increased cell proliferation and metastasis [28]. Therefore, the activation of the WNT/β-catenin pathway promotes cancer. Through GSEA, we showed that KRT17 was significantly involved in several important keystone pathways, the most important being the WNT-related pathways.

In COAD, APC loss or mutation is the main driving factor for the activation of the WNT signaling pathway [29]. Tumors with truncated mutations in the APC gene also show high expression of β-catenin [30]. As a gene with protective function, the loss of APC causes increased COAD incidence. The increased co-expression of KITENIN and ErbB4-CYT-2 promotes the transition from colonic adenoma to adenocarcinoma in the tumor microenvironment associated with APC loss [31, 32]. Correspondingly, when its function is restored, it results in rapid and extensive differentiation and regression of tumor cells for its inhibition [32]. Through STRING analysis, we identified KRT17 interaction with APC; when the expression of APC increased, the expression of KRT17 was inhibited. When APC was overexpressed in SW116 cells with high KRT17 expression, tumor cell invasion and metastasis were inhibited to a certain extent, while the expression of β-catenin was significantly lowered. This phenomenon was reversed upon Wnt 3a exposure, an activator of the WNT signaling pathway.

Previous studies have shown that the expression of KRT17 is related to the tumor progression and poor prognosis of gastric adenocarcinoma [14]. However, due to the limitation of the patient follow-up period and the limited number of clinical samples collected, we did not conduct a more in-depth study on the prognosis of patients with colon adenocarcinoma by KRT17. In our follow-up studies, we will continue to explore how APC affects the WNT/β-catenin signaling pathway involved in the regulation of KRT17.

## 5. Conclusion

In summary, our research proved that the overexpression of KRT17 promoted COAD progress. It could activate WNT/β-catenin-related pathways, which in turn led to enhanced tumor metastasis and invasion. However, these effects were suppressed upon APC overexpression. In addition, the increased expression of KRT17 played a role in promoting the formation of new blood vessels. Therefore, we speculated that the increased expression of KRT17 in COAD could worsen the outcome of COAD. Taken together, our data provided convincing new evidence, which indicated that KRT17 could be a promising new direction for the development of COAD-targeted immunotherapy.

## Supplementary Material

Supplemental Material

## Data Availability

The raw data supporting the conclusions of this manuscript will be made available by the authors, without undue reservation, upon request to any qualified researcher. The data sources in the manuscript are as follows:TCGA:https://portal.gdc.cancer.gov/;string:https://cn.string-db.org/;GSEA:http://www.gsea-msigdb.org/gsea/index.js

## Research highlights

- Keratin 17 was highly expressed in colon adenocarcinoma tissues.
- Keratin 17 was significantly associated with metastasis and angiogenesis of colon cancer cells.
- Keratin 17 promotes the development of COAD by regulating WNT/β-catenin signaling pathway.
