# Correction

**DOI:** 10.1080/21655979.2026.2719357

**Published:** 2026-08-26

**Article title**: Keratin 17 upregulation promotes cell metastasis and angiogenesis in colon adenocarcinoma.

**Authors**: Ji, R., Ji, Y., Ma, L., Ge, S., Chen, J., Wu, S., Huang, T., Sheng, Y., Wang, L., Yi, N., Liu, Z.

**Journal**: *Bioengineered*

**Bibliometrics**: *12(2), 12598–12611*

**DOI**: 10.1080/21655979.2021.2010393

The supplementary file https://doi.org/10.6084/m9.figshare.17372257.v1 has been removed as it was in breach of the Publisher Editorial Policies.

