# Supplementary material for: Keratin 17 upregulation promotes cell metastasis and angiogenesis in colon adenocarcinoma
Source: Bioengineered. 2021 Dec 22;12(2):12598–611. doi: 10.1080/21655979.2021.2010393 (PMC8809968; doi:10.1080/21655979.2021.2010393)
Supplement: Supplemental Material [file KBIE_A_2010393_SM9852.zip › supplementary/supplementary materials.docx]

***Materials and Methods***

*1.Inmunohistochemistry (IHC)*

Samples were processed for immunohistochemical analysis to determine the KRT17 and CD31 expression levels and their localization. Rabbit polyclonal antibodies KRT17 (1:500, Proteintech, China) and CD31 (1:200, Proteintech, China) were used for detection. The antigen-antibody complex was visualized using diaminobenzidine (DAB, 5 minutes incubation) and counterstained with hematoxylin. PBS was used as the negative control. KRT17 immunoreactivity was detected in the cytoplasm of the carcinoma cells, CD31 was detected in the neovascularization, and the sample sections were scored semi-quantitatively for immunoreactivity as follows: 0 = 0% stained; 1 = 1–49% stained; 2 = 50–100% stained immunoreactive cells. Additionally, the intensity of staining was scored semi-quantitatively as 0, negative; 1, weak; 2, intermediate; and 3, strong. The final immunoreaction score was defined as the sum of both extent and intensity parameters. Final immunoreaction scores >0 were defined as positive. Photographs were observed under an optical microscope. Two experienced pathologists independently evaluated the slices.

*2.Immunofluorescence staining*

To determine the localization of β-catenin and KRT17, cells were seeded on coverslips and fixed using 4% paraformaldehyde for 40 min. Coverslips were rinsed with PBS, and cells were permeabilized with 0.1% Triton X-100 for 20 min. Following PBST washes thrice, the cells were blocked using 1% BSA at 4℃ for two hours. Subsequently, the cells were incubated with anti-β-catenin antibody (1:200, Proteintech, China) and anti-KRT17 antibody overnight at 4℃. After washing with PBST, cells were incubated with rhodamine-labeled goat anti-rabbit secondary antibody (1:200, Sigma, USA) for 1 h and 40,6-Diamidino-2-phenylindole (DAPI; Invitrogen, USA) was used to stain the nucleus. Images were captured using the OLYMPUS U-RFL-T upright fluorescence microscope.

*3.Nuclear and Cytoplasmic Protein Extraction*

After the appropriate treatment, the nuclear and cytoplasmic fractions of proteins were extracted from the cells. Briefly, cells were treated with 250 μL extraction buffer (10 mmol/L Tris-HCl, 10 mmol/L KCl, and 5 mmol/L MgCl2; pH 7.6). Then, to disrupt the cell membrane, cells were incubated with 0.5% Triton X-100 for 40 min. 250 μL of nuclear isolation buffer (10 mmol/L Tris-HCl, 10 mmol/L KCl, 5 mmol/L MgCl2, and 0.35 mol/L sucrose) was subsequently added, and the sample was subjected to density gradient centrifugation for 10 min. The supernatant contained the cytoplasmic fraction and the precipitate contained the nuclear fraction. The supernatant was transferred to a fresh centrifuge tube, and four volumes of pre-chilled acetone were added to it at -20℃ and incubated overnight. Next, the supernatant was centrifuged at 12, 000 rpm for 20 min at 4℃. The precipitate was dissolved in SDS buffer and centrifuged at 12, 000 rpm for 30 min at 4℃. After centrifugation, the supernatant containing the nuclear proteins was collected.

***Supplementary figure：***

**Supplementary figure 1**


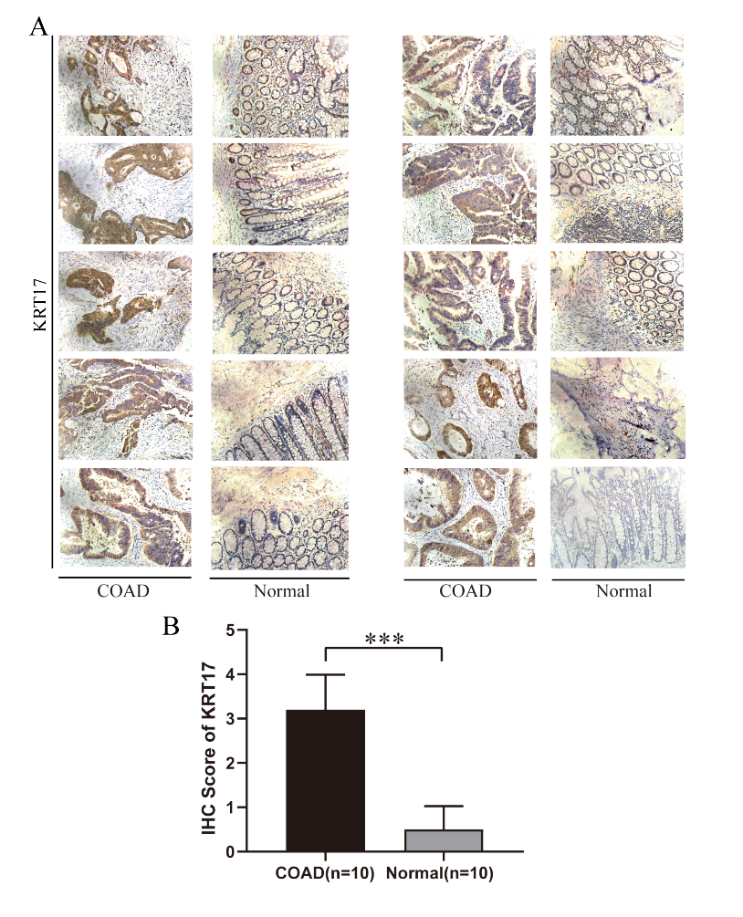


**Supplementary figure 2**


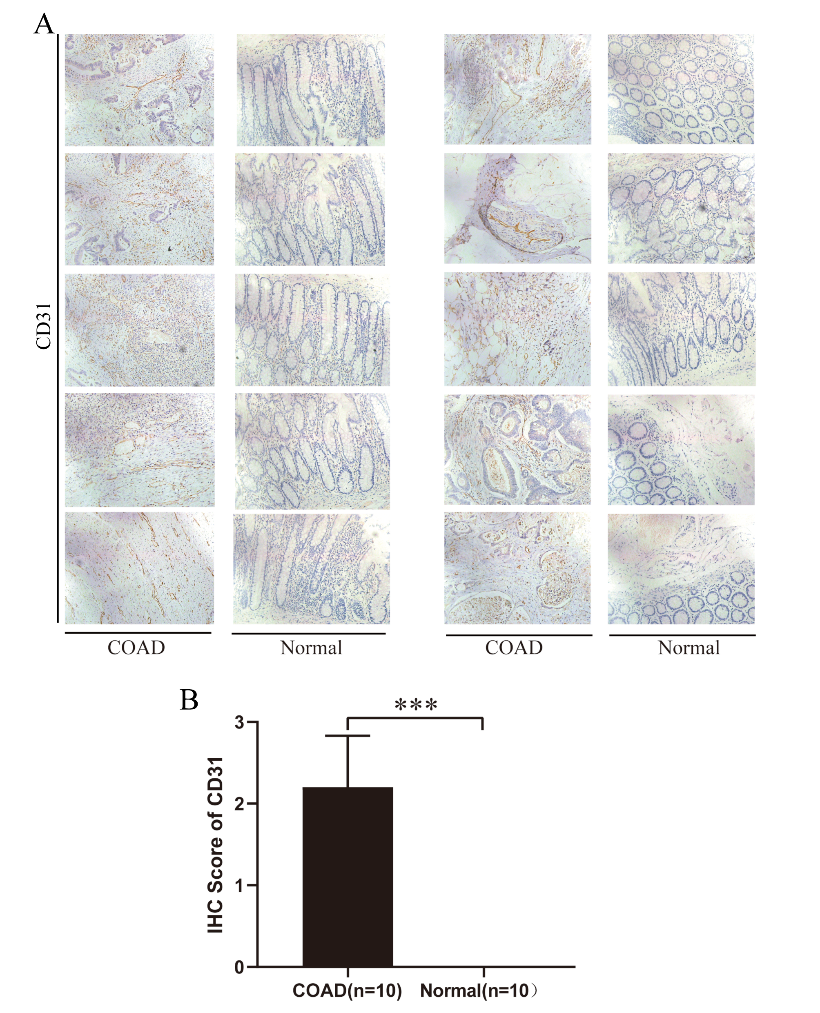


**Supplementary figure 3**


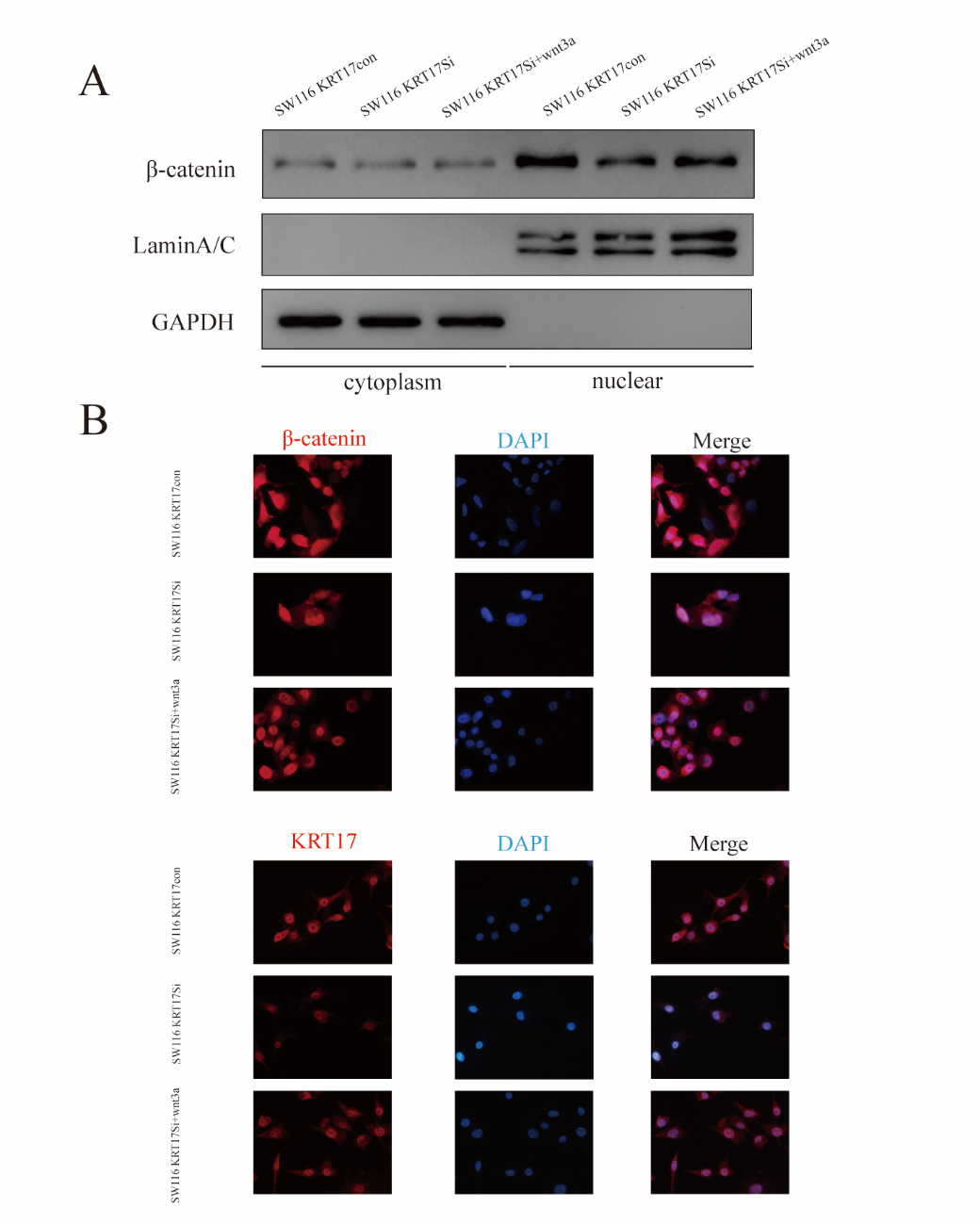


***Figure legend：***

**Supplement figure 1**: The expression of KRT17 in 10 pairs of COAD tissue and corresponding normal colonic tissue**.(A)** KRT17 immunohistochemistry of 10 pairs of COAD tissue and its corresponding normal colonic epithelial tissue. **(B)** IHC score of KRT17(***P＜0.001 according to the two-tailed Student’s t-test).

**Supplement figure 2**: The expression of CD31 in 10 pairs of COAD tissue and corresponding normal colonic tissue.**(A)** CD31 immunohistochemistry of 10 pairs of COAD tissue and its corresponding normal colonic epithelial tissue. **(B)** IHC score of CD31(***P＜0.001 according to the two-tailed Student’s t-test).

**Supplement figure 3**: β-catenin is distributed in the nucleus of SW116 cells.**(A)**Western blot analysis of cytoplasmic and nuclear distribution of β-catenin. GAPDH and LaminA/C in cells were used as specific markers for cytoplasmic and nuclear components, respectively.**(B)**Immunofluorescence analysis of β-catenin and KRT17 in SW116 KRT17 Con cells and SW116 KRT17 si cells with or without Wnt3a (2 μM) pretreatment.
