# Supplementary figures and images for: Keratin 17 upregulation promotes cell metastasis and angiogenesis in colon adenocarcinoma
Source: Bioengineered. 2021 Dec 22;12(2):12598–611. doi: 10.1080/21655979.2021.2010393 (PMC8809968; doi:10.1080/21655979.2021.2010393)

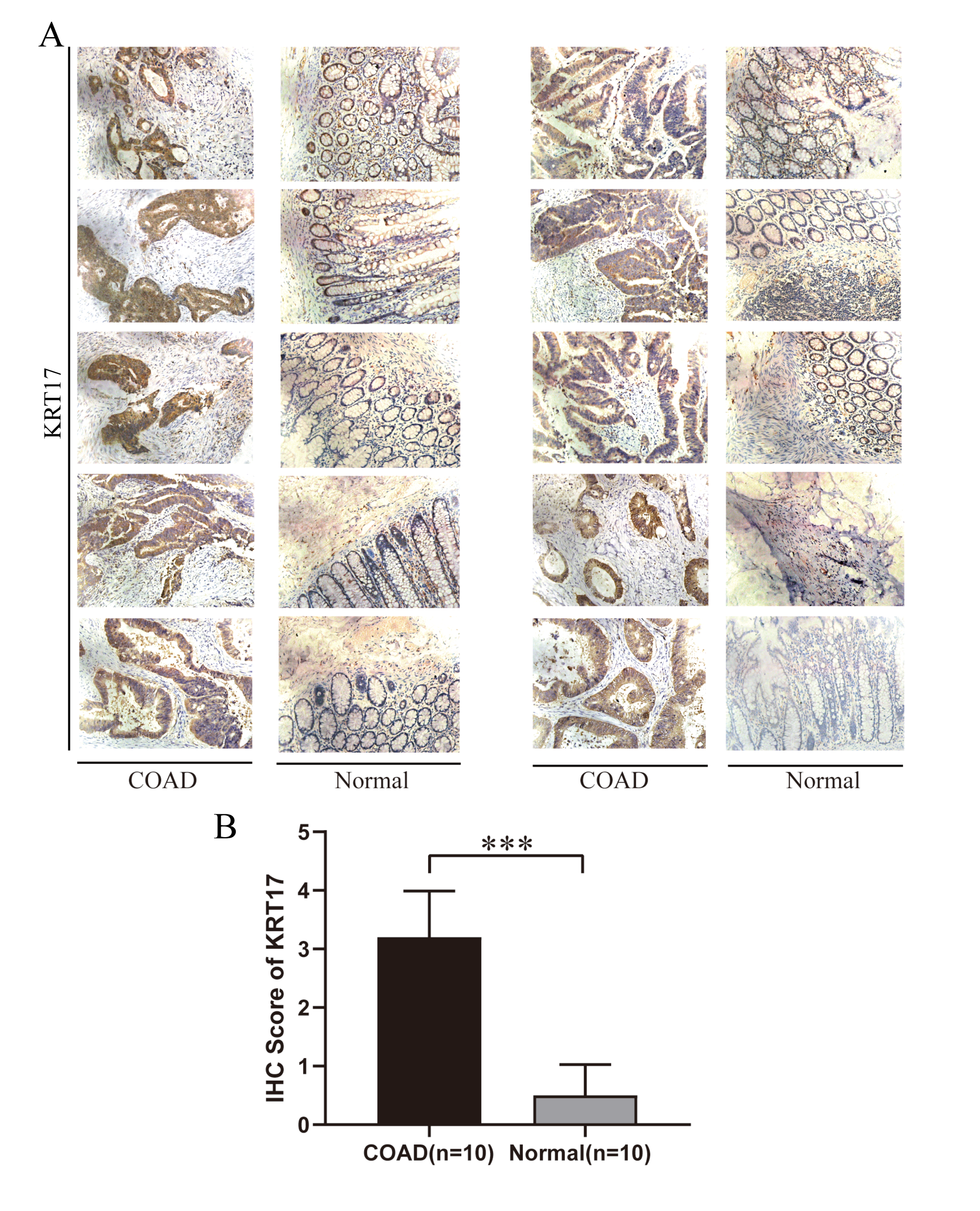

Supplement: Supplemental Material [file KBIE_A_2010393_SM9852.zip › supplementary/supplement figure 1.tif]

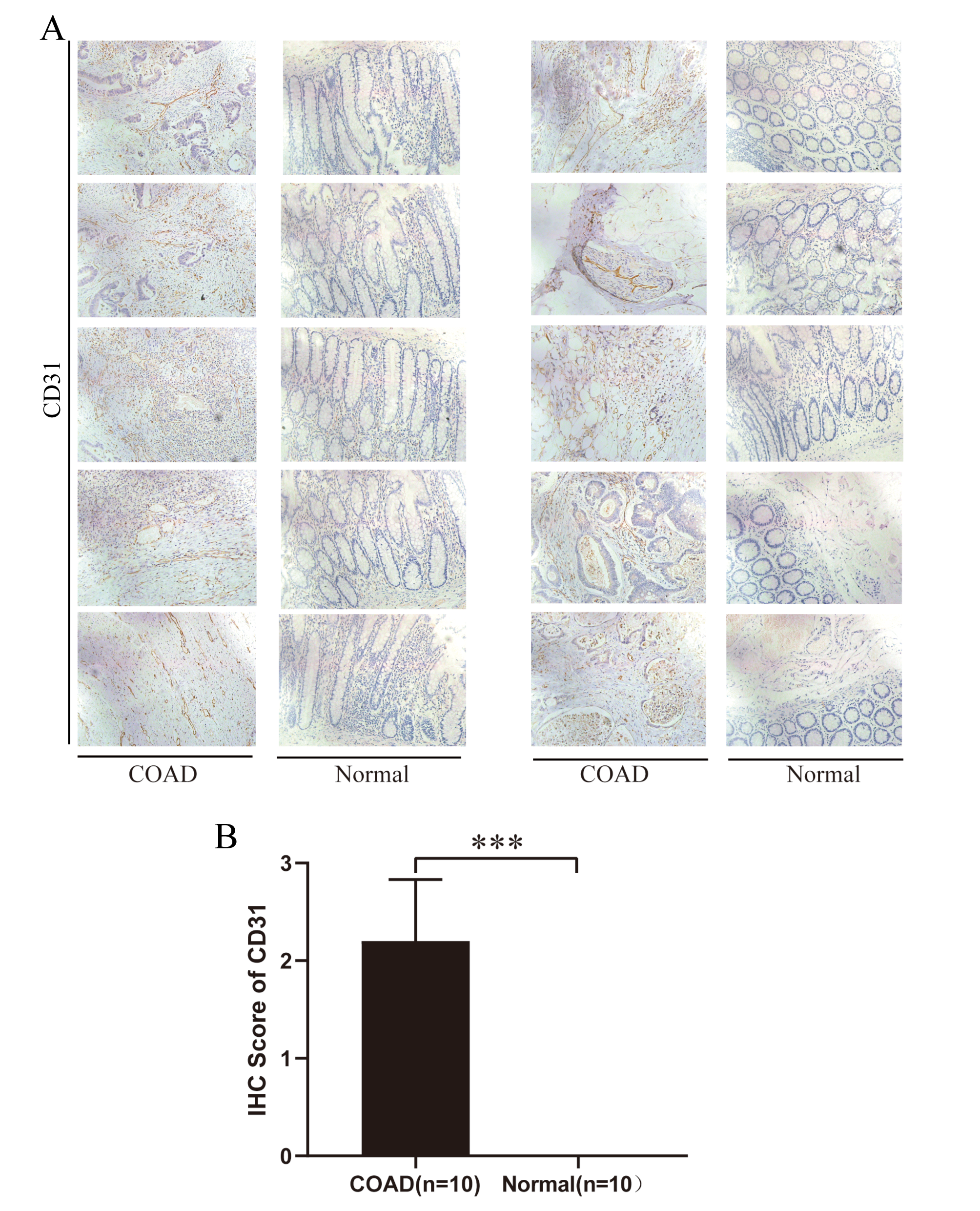

Supplement: Supplemental Material [file KBIE_A_2010393_SM9852.zip › supplementary/supplement figure 2.tif]

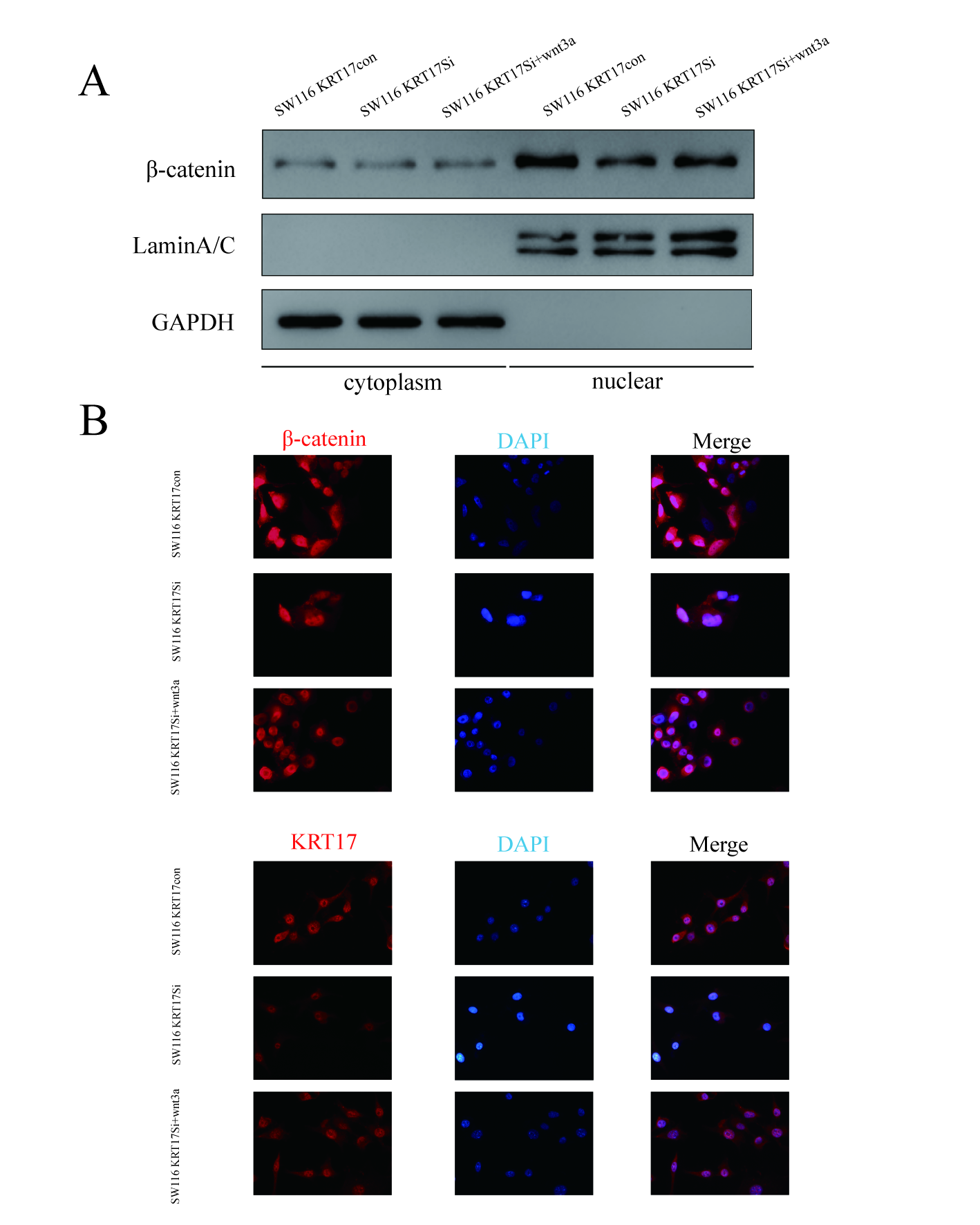

Supplement: Supplemental Material [file KBIE_A_2010393_SM9852.zip › supplementary/supplement figure 3.tif]
